# An Average Body Circumference Can Be a Substitute for Body Mass Index in Women

**DOI:** 10.1155/2014/592642

**Published:** 2014-11-19

**Authors:** Antonis Polymeris, Peter D. Papapetrou, Georgios Katsoulis

**Affiliations:** Second Division of Endocrinology, Alexandra Hospital, 11528 Athens, Greece

## Abstract

*Introduction.* Significant correlations between BMI and some body circumferences have been previously reported. In this study we investigated if the average of the sum of eight body circumferences can be a substitute for BMI. *Patients and Methods.* BMI and eight body circumferences (neck, waist, hip, arm, forearm, wrist, thigh, and ankle) were measured in 193 apparently healthy women aged 20–83, and within a wide range of BMI. Women with BMI ≤ 24.9 were designated as normal, with BMI 25–29.9 as overweight and with BMI ≥ 30 as obese. The relationship of the average body circumference (ABC) of the sum of the eight circumferences, and of each individual circumference with BMI, was evaluated. *Results.* ABC had the strongest correlation with BMI (*r* = 0.95, *P* < 0.001) among all the circumferences tested. Hip circumference had the strongest correlation with BMI (*r* = 0.89, *P* < 0.001) among the circumferences of individual body sites. Receiver-Operator Characteristic analysis showed that women with ABC > 44.0 cm could be recognized as having BMI ≥ 25 with sensitivity 90.2% and specificity 88.5%, while women with ABC > 47.1 cm could be diagnosed as having BMI ≥ 30 with sensitivity 92.2% and specificity 91.5%. *Conclusion.* An average body circumference strongly correlated with BMI in women and can serve as a surrogate of BMI.

## 1. Introduction

Body mass index (BMI) has been used extensively as a convenient and inexpensive measure of obesity in epidemiological studies since its introduction in 1972 [1]. In a considerable number of studies, high BMI has been associated with increased cardiovascular morbidity and mortality, resistance to insulin, and diabetes mellitus [2]. However, while BMI is appropriate for population studies, it should not be used as an index of obesity in individual patients [1], but this is a standard practice by many clinicians and is recommended by health authorities as a screening test for obesity [3–5]. A major shortcoming of BMI is that it does not give any estimation of the distribution of fat in the human body. Moreover, for the estimation of BMI, special equipment for the measurement of body weight and height is needed. Ben-Noun et al. [6] found a significant correlation between neck circumference and BMI and proposed that neck circumference could be used to identify BMI-defined overweight or obese patients. In the present work, we investigated the relationship between BMI and several body circumferences in women and we found that the best predictor of BMI among various body circumferences is the average of the sum of eight individual body circumferences (ABC).

## 2. Participants and Methods

The study population included 193 apparently healthy women aged 20–83 (60.0 ± 13.6, mean ± SD). This population consisted of some women from the hospital personnel and some of their relatives, as well as some women who referred to the endocrine clinic of the hospital for evaluation of their thyroid function, and some of their healthy relatives. Only euthyroid women without significant goiter and those with microscopic thyroid nodules not affecting the neck circumference were included. Diabetic patients and women with somatic deformities were excluded. The participant women were categorized in three subgroups as shown in Table 1: those with BMI ≤ 24.9 designated as “normal,” with BMI 25–29.9 (overweight), and with BMI ≥ 30 (obese). The study was approved by the Scientific Committee of the Alexandra Hospital and the data were collected with the participant's informed consent.

Body weight and height were measured with standard techniques in the women wearing light indoor clothing. Body weight was recorded to the nearest 0.1 kg and height to the nearest 0.5 cm. BMI was calculated as weight in kg divided by the square of height in meters. Body circumferences were measured using a flexible cloth tape measure. The measurement of the body circumferences was performed with the individual standing and looking straight ahead, with her legs in loose contact and her arms hanging close to her body. The neck circumference was measured just above the cricoid cartilage, the waist circumference at the middle of the distance between the lower rib and the iliac crest, the hip circumference around the widest portion of the buttocks, the distal thigh circumference ten centimeters above the upper edge of the patella, the ankle circumference at the narrowest point of the region, the arm circumference at the middle of the distance between the acromion and olecranon process, the circumference of the forearm at the widest portion of the forearm, and the wrist circumference around the bony part of the wrist.

Eight individual body circumferences were measured and the averages of several different sums of individual body circumferences were also calculated. Average body circumference (ABC) is the average of the sum of the eight circumferences (neck, waist, hip, arm, forearm, wrist, thigh, and ankle). Upper body circumference (UBC) is the average of the sum of neck, waist, arm, forearm, and wrist. Lower body circumference (LBC) is the average of the sum of hip, thigh, and ankle.

## 3. Statistical Analysis

A Kolmogorov-Smirnov normality test showed that some of the parameters depicted in Table 1 had nonnormal distribution and therefore the geometric means and 95% CI (confidence interval) are presented. Because of nonnormality of the variables, simple Spearman Rank correlations were performed between each circumference and BMI. A Receiver-Operator Characteristic (ROC) analysis was performed in order to evaluate the diagnostic ability of body circumferences to recognize persons within certain ranges of BMI. The statistical analysis was performed using GraphPad Prism Software, San Diego, CA, USA.

## 4. Results

Anthropometric characteristics and the geometric means (95% CI) of some parameters for the entire study population and for three BMI stratified subpopulations are shown in Table 1.

### 4.1. Relationship between BMI and Individual Body Circumferences

Hip circumference showed the strongest simple correlation with BMI among the circumferences of individual body sites (*r* = 0.89, *P* < 0.001). Average values of various sums of body circumferences showed generally stronger simple correlations with BMI when compared with circumferences of individual body sites; thus, the ABC had the strongest correlation with BMI among all the circumferences tested (*r* = 0.95, *P* < 0.001) (Table 2).

### 4.2. Relationship of BMI with ABC, UBC, and LBC

**Figure 1:**
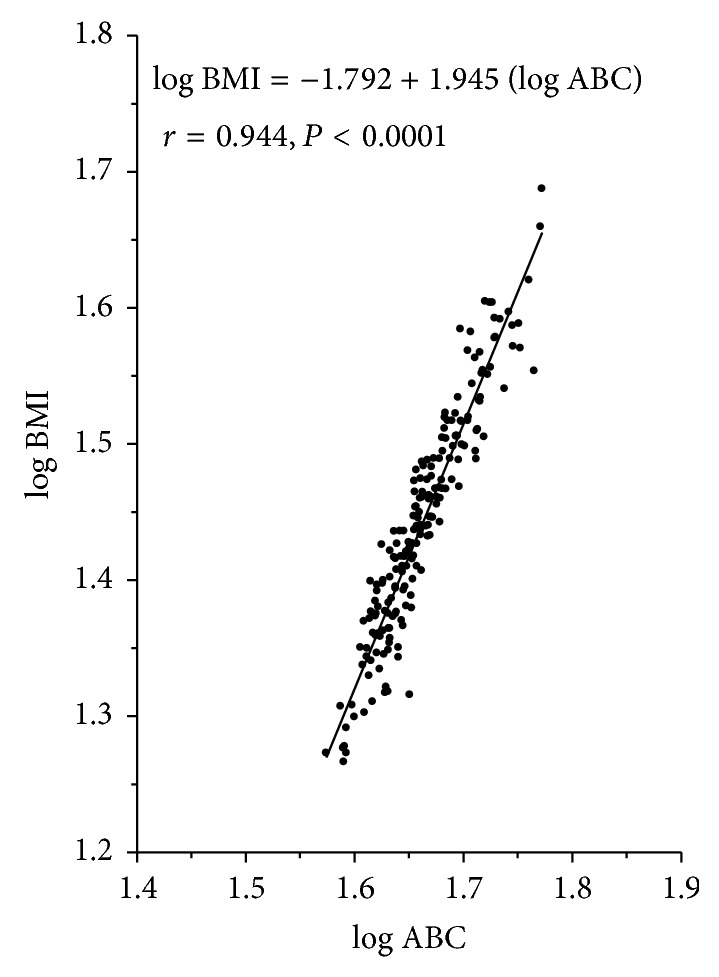
Linear regression of log ABC (average body circumference) on log BMI in the entire study population.

The log transformed values of the BMI and ABC values in the entire study population showed normal distribution. Simple linear regression of log BMI on log ABC showed a strong correlation between these two parameters in the entire study population (*r* = 0.94, *P* < 0.001); the regression coefficient *r* ^2^ = 0.89 implies that 89% of the variation of BMI may be explained by ABC (Figure 1). In order to adjust for age, log BMI was regressed simultaneously on log ABC and age whose distribution was not far from normal. Age did not significantly affect the effect of ABC on BMI (beta coefficient for ABC 0.93, and for age −0.09). Simple correlation of BMI with ABC (*r* = 0.95) was slightly higher when compared with that between BMI and UBC (*r* = 0.91) or that between BMI and LBC (*r* = 0.89) (Table 2). A similar trend was observed more or less in the three subgroups.

In the entire study population, ABC was highly correlated with body weight (*r* = 0.94, *P* < 0.001) but not with height (Table 3). A strong simple correlation was also found between ABC and body weight but not with height in all the subgroups, except in the overweight women in whom ABC was significantly correlated with body weight as well as with height (Table 3).

### 4.3. Receiver-Operator Characteristic Analysis

**Figure 2:**
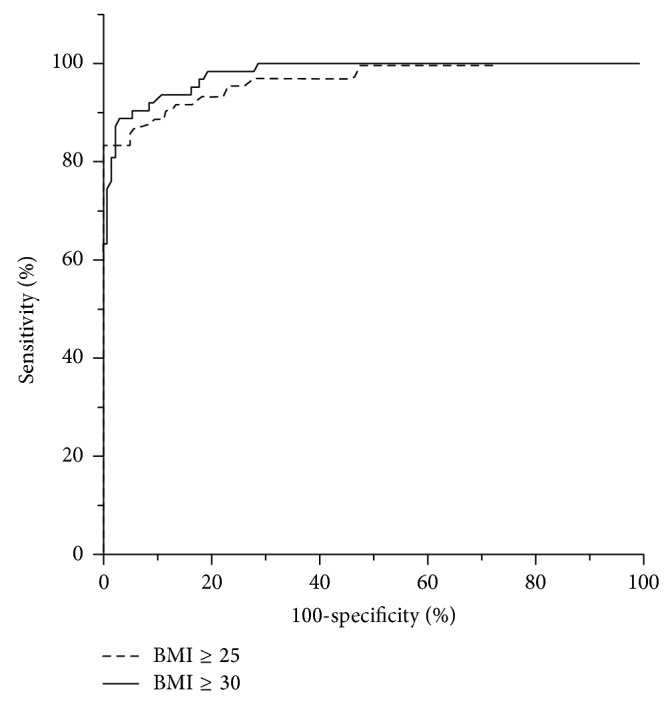
ROC curves for the recognition of women with BMI ≥ 25 or BMI ≥ 30 using the average body circumference (ABC). The area under the BMI ≥ 25 curve is 0.97 and that under the BMI ≥ 30 curve is 0.98.

In order to test if persons with BMI ≥ 25 or with BMI ≥ 30 may be recognized using a body circumference, Receiver-Operator Characteristic (ROC) analysis was performed. The results are summarized in Table 4. The best performance in that regard had the ABC. Thus, for ABC, the area under the ROC curve had the value 0.97 (0.94–0.99, 95% CI) for the BMI ≥ 25 ROC curve and 0.98 (0.96–1.0) for the BMI ≥ 30 ROC curve (Figure 2). Women with ABC > 44.0 cm could be recognized as having BMI ≥ 25 with sensitivity 90.2% and specificity 88.5%. Women with ABC > 47.1 cm could be recognized as having BMI ≥ 30 with sensitivity 92.2% and specificity 91.5% (Table 4).

## 5. Discussion

The purpose of the present work was to evaluate the relationship of various body circumferences with BMI and determine whether any circumference could be used to identify BMI-defined overweight and obese patients as was shown previously for the neck circumference [6].

In the present study, we assessed the relationship between BMI and each of eight individual body circumferences and also between BMI and the average value of several sums of individual circumferences. The correlation between BMI and the averaged sums of individual circumferences was generally stronger than that between BMI and any of the individual body circumferences (Table 2). Thus, in the entire study population, BMI was strongly correlated with the average body circumference (ABC) with regression coefficient *R* ^2^ = 0.89, which implies that 89% of the variation of BMI is due to ABC (Table 2). In the female participants in our study, we found a strong positive correlation between BMI and ABC, between ABC and body weight, and between BMI and body weight as expected and a significant but weaker correlation between body weight and height. There was not significant correlation between BMI and height or between ABC and height. These results taken together imply that body weight depends much more on the average body circumference than on height and that the strong relationship of ABC with BMI may be explained mainly by the strong relationship between ABC and body weight.

The measurement of BMI (or of body weight) cannot differentiate adipose from lean body mass. Therefore, in slim-bodied persons (presumably with normal BMI), BMI reflects more the lean body mass than in persons with excess adipose tissue (with high BMI). This is a reason for the poor diagnostic accuracy of BMI to diagnose obesity as measured by other direct methods, especially for persons in the intermediate BMI ranges [7–10]. An upper body fat distribution is considered to have generally a worse prognosis when compared to a lower body fat distribution [11]. It would be therefore of interest to compare the relationship of upper body circumference (UBC) with BMI to that of lower body circumference (LBC). Thus, in the entire study population, simple correlation of BMI with the UBC (*R* ^2^ = 0.83) and with the LBC (*R* ^2^ = 0.79) showed that 83% of the variation of BMI is due to the UBC and 79% is due to the LBC. In contrast, in the overweight women, 48% of the variation of the BMI may be explained by UBC compared to only 24% explained by LBC, while, in the obese women, UBC and LBC explain practically the same percentage of variation (45% and 42% resp.), and the same applies to normal women in whom 29% of the variation of BMI is explained by UBC and 24% by LBC (Table 2). Among the three BMI stratified subgroups, only in the overweight a positive correlation was found between waist/hip ratio and BMI. These data imply that women in the intermediate range of BMI tend to accumulate fat preferably in the waist when gaining weight.

ROC analysis showed that ABC can identify BMI-defined overweight or obese women with very good sensitivity and specificity (Table 4, Figure 2). However, in terms of sensitivity and specificity, the performance of some other body circumferences was practically not inferior to that of ABC (Table 4). Also, UBC and LBC in their ability to identify overweight or obese women were practically not different.

Heymsfield et al. [12] studied the relationship between BMI and five individual circumferences (arm, waist, hip, thigh, and calf) and between BMI and the average of the sum of these five circumferences using analytical methods different from the methods used in the present study. Our findings agree with the results of these authors who found that BMI (represented by body volume/height ratio) was strongly correlated with the average as well as with each of the individual circumferences [12]. In our study, we included neck circumference because several studies showed this circumference to be a useful anthropometric measure of obesity [6, 13, 14]. In the present study, we considered also wrist and ankle circumferences representing regions where it is less likely for fat to accumulate in obesity.

The following conclusions may be drawn from the results of our study. In a population of women with a wide range of BMI, the average of the sum of eight body circumferences (ABC) had a very strong correlation with BMI; however, the average of some other more convenient sums of circumferences (as shown in Table 2) had almost equally strong correlations with BMI. The degree of correlation between individual circumferences and BMI was generally smaller than that between averaged sums of circumferences and BMI. Among individual circumferences, the hip and the waist had the strongest and practically not different correlation with BMI. ROC analysis demonstrated that an ABC circumference can identify women with BMI ≥ 25 or with BMI ≥ 30 with very good sensitivity and specificity. Measurement of body circumferences is simple, does not need special equipment for the measurement of body weight and height, and therefore may be especially suitable for field epidemiological studies. The time required for the measurement of the eight body circumferences was approximately one minute comparing favorably with the time required for the measurement of weight and height. It should be noted that an accurate measurement of height may be a time-consuming procedure. Average body circumference can be a surrogate of BMI.

## Figures and Tables

**Table 1:** Anthropometric parameters of the study population.

Participants	All (*n* = 193)	Normal (*n* = 61)	Overweight (*n* = 68)	Obese (*n* = 64)
Parameter				
Age	48.9 (46.8–51.0)	44.5 (41.2–48.1)	52.9 (49.8–56.1)	49.1 (45.0–53.7)
Weight (kg)	71.1 (69.2–73.1)	58.3 (56.9–59.7)	69.5 (67.9–71.0)	88.1 (85.3–91.1)
Height (cm)	160.0 (159.2–160.8)	161.4 (160.1–162.6)	159.1 (157.8–160.4)	159.7 (157.9–161.5)
BMI	27.8 (27.0–28.6)	22.4 (21.9–22.9)	27.5 (27.1–27.8)	34.6 (33.7–35.5)
Circumference of (cm)				
Neck	33.8 (33.5–34.1)	31.9 (31.5–32.2)	33.6 (33.3–34.0)	35.9 (35.4–36.5)
Waist	91.1 (89.3–92.9)	79.8 (78.0–81.7)	89.6 (87.9–91.4)	105.0 (102.4–107.6)
Hip	107.1 (105.6–108.6)	97.8 (96.7–98.9)	105.0 (103.8–106.3)	119.2 (116.8–121.6)
Arm	26.5 (26.1–27.0)	23.8 (23.5–24.2)	26.5 (26.1–26.9)	29.4 (28.7–30.2)
Forearm	24.90 (24.6–25.2)	23.1 (22.8–23.5)	24.8 (24.5–25.1)	26.8 (26.3–27.5)
Wrist	16.0 (15.8–16.2)	15.1 (14.9–15.4)	15.9 (15.7–16.2)	16.9 (16.5–17.2)
Thigh	45.3 (44.6–46.1)	41.5 (40.6–42.4)	44.4 (43.6–45.3)	50.4 (49.2–51.7)
Ankle	23.2 (22.9–23.5)	21.8 (21.4–22.2)	23.1 (22.7–23.5)	24.8 (24.3–25.4)
Waist/hip ratio	0.85 (0.84–0.86)	0.82 (0.80–0.83)	0.85 (0.84–0.87)	0.88 (0.86–0.90)
Mean circumference of (cm)				
ABC^a^	46.1 (45.5–46.7)	41.9 (41.5–42.4)	45.4 (45.0–45.9)	51.2 (50.4–52.0)
UBC^b^	38.5 (38.0–39.1)	34.8 (34.3–35.3)	38.1 (37.7–38.6)	42.9 (42.1–43.6)
LBC^c^	58.6 (57.8–59.4)	53.8 (53.2–54.4)	57.6 (56.9–58.2)	64.9 (63.7–66.1)
(N + W + H + A + Th)/5	60.9 (60.0–61.7)	55.0 (54.4–55.7)	59.9 (59.3–60.5)	68.1 (67.0–69.2)
(Waist + hip + arm + thigh)/4	68.3 (67.1–69.6)	60.8 (60.1–61.6)	66.5 (65.8–67.2)	76.1 (74.8–77.5)
(Neck + waist + hip)/3	77.4 (76.3–78.6)	69.9 (69.0–70.8)	76.2 (75.4–77.0)	86.8 (85.3–88.3)
(Waist + hip)/2	99.2 (97.6–100.8)	88.9 (87.6–90.2)	97.4 (96.3–98.6)	112.2 (110.2–114.3)

Normal = women with BMI ≤24.9. Overweight = women with BMI 25–29.9. Obese = women with BMI ≥30.

^a^ABC, average body circumference, average of (neck + waist + hip + arm + forearm + wrist + thigh + ankle).

^b^UBC, upper body circumference, average of (neck + waist + arm + forearm + wrist).

^c^LBC, lower body circumference, average of (hip + thigh + ankle).

N = neck, W = waist, H = hip, A = arm, F = forearm, Wr = wrist, Th = thigh, and Ank = ankle. Geometric means (95% CI) are presented.

**Table 2:** Spearman Rank correlations of BMI with body circumferences.

Participants	All (*n* = 193)	Normal (*n* = 61)	Overweight (*n* = 68)	Obese (*n* = 64)
	*R*	*R*	*R*	*R*
*BMI with circumference of *				
Hip	0.89	0.56	0.53	0.61
Waist	0.84	0.49	0.57	0.56
Arm	0.82	0.42	0.47	0.49
Forearm	0.79	0.52	0.42	0.57
Neck	0.77	0.48	0.32	0.54
Thigh	0.73	0.45	0.31	0.50
Wrist	0.61	0.30^**^	0.29	0.38
Ankle	0.61	0.39	0.22^*^	0.44
Waist/hip ratio	0.40	0.08^*^	0.28^**^	0.10^*^
ABC^a^	0.95	0.60	0.77	0.80
UBC^b^	0.91	0.54	0.69	0.67
LBC^c^	0.89	0.49	0.49	0.65
(N + W + H + A + Th)/5	0.95	0.58	0.77	0.78
(W + H + A + Th)/4	0.94	0.57	0.75	0.77
(Neck + waist + hip)/3	0.93	0.55	0.72	0.73
(Waist + hip)/2	0.92	0.54	0.70	0.71

Normal, with BMI ≤24.9. Overweight, with BMI 25–29.9. Obese, with BMI ≥30.

^a^ABC = average body circumference, average of (neck + waist + hip + arm + forearm + wrist + thigh + ankle).

^b^UBC, upper body circumference, average of (neck + waist + arm + forearm + wrist).

^c^LBC, lower body circumference, average of (hip + thigh + Ankle). N = neck, W = waist, H = Hip, A = arm, and Th = thigh.

The coefficients (*R*) marked with one asterisk were nonsignificant, with two asterisks having *P* = 0.02, and the rest without asterisk had *P* ≤ 0.001.

**Table 3:** Spearman Rank intercorrelations between BMI, body weight, height, and average body circumference (ABC).

Participants	All (*n* = 193)	Normal (*n* = 61)	Overweight (*n* = 68)	Obese (*n* = 64)
	*R*	*R*	*R*	*R*
*Correlation of *				
BMI with				
Body weight	0.93	0.73	0.69	0.69
Height	−0.12^*^	−0.11^*^	0.16^*^	−0.08^*^
ABC^a^	0.95	0.60	0.77	0.80
Body weight with				
Height	0.22	0.56	0.80	0.63
ABC^a^	0.94	0.63	0.76	0.82
ABC^a^ with				
Height	0.03^*^	0.19^*^	0.40	0.29^**^

Normal, with BMI ≤24.9, overweight, with BMI 25–29.9, and obese, with BMI ≥30.

^a^ABC, average body circumference, average of (neck + waist + hip + arm + forearm + wrist + thigh+ ankle).

The coefficients (*R*) with one asterisk were not significant, with two asterisks having *P* = 0.03, and the rest without asterisk had *P* < 0.001.

**Table:** (a) Recognition of women with BMI ≥25. (prevalence of BMI ≥25: 68.4%)

Body Circumference	Area under the ROC curve	Cutoff value (cm)	Sensitivity (%)	Specificity (%)
ABC	0.97 (0.94–0.99)	>44.0	90.2	88.5
UBC	0.94 (0.91–0.94)	>36.7	87.9	86.9
LBC	0.93 (0.89–0.96)	>56.3	84.9	82.0
(N + W + H + A + Th)/5	0.96 (0.94–0.98)	>58.0	90.2	86.9
(W + H + A + Th)/4	0.96 (0.93–0.98)	>63.9	90.9	85.3
Neck	0.88 (0.83–0.93)	>32.6	87.1	75.4
Waist	0.90 (0.86–0.95)	>83.9	90.2	73.8
Hip	0.93 (0.89–0.96)	>101.4	90.2	80.3

**Table:** (b) Recognition of women with BMI ≥30. (prevalence of BMI ≥30: 33.2%)

Body Circumference	Area under the ROC curve	Cutoff value (cm)	Sensitivity (%)	Specificity (%)
ABC	0.98 (0.96–0.99)	>47.1	92.2	91.5
UBC	0.95 (0.92–0.98)	>39.2	92.2	85.3
LBC	0.96 (0.93–0.99)	>59.5	90.6	87.6
(N + W + H + A + Th)/5	0.98 (0.97–1.0)	>62.5	93.8	91.5
(W + H + A + Th)/4	0.98 (0.97–0.99)	>69.6	93.8	91.5
Neck	0.88 (0.83–0.93)	>34.0	87.5	73.6
Waist	0.93 (0.89–0.96)	>90.6	92.2	76.7
Hip	0.96 (0.94–0.99)	>109.8	90.6	89.2

ABC, average body circumference, average of (neck + waist + hip + arm + forearm + wrist + thigh + ankle).

UBC, upper body circumference, average of (neck + waist + arm + forearm + wrist).

LBC, lower body circumference, average of (hip + thigh + ankle).

N = neck, W = waist, H = hip, A = arm, and Th = thigh. Area under the ROC curve (95% CI). Cutoff value of body circumferences.

## Abbreviations

BMI: Body mass index
ABC: Average body circumference, average of (neck + waist + hip + arm + forearm + wrist + thigh + ankle)
UBC: Upper body circumference, average of (neck + waist + arm + forearm + wrist)
LBC: Lower body circumference, average of (hip + thigh + ankle)
ROC analysis: Receiver-operator characteristic analysis.
